# Survival associated pathway identification with group *L_p _*penalized global AUC maximization

**DOI:** 10.1186/1748-7188-5-30

**Published:** 2010-08-16

**Authors:** Zhenqiu Liu, Laurence S Magder, Terry Hyslop, Li Mao

**Affiliations:** 1 Greenebaum Cancer Center, University of Maryland, 22 South Greene Street, Baltimore, MD 21201, USA; 2 Department of Epidemiology and Preventive Medicine, The University of Maryland, Baltimore, MD 21201, USA; 3 Division of Biostatistics, Department of Pharmacology and Experimental Therapeutics, Thomas Jefferson University, Philadelphia, PA 19107, USA; 4 Department of Oncology and Diagnosis Sciences, The University of Maryland Dental School, Baltimore, MD 21201, USA

## Abstract

It has been demonstrated that genes in a cell do not act independently. They interact with one another to complete certain biological processes or to implement certain molecular functions. How to incorporate biological pathways or functional groups into the model and identify survival associated gene pathways is still a challenging problem. In this paper, we propose a novel iterative gradient based method for survival analysis with group *L_p _*penalized global AUC summary maximization. Unlike LASSO, *L_p _*(*p *< 1) (with its special implementation entitled adaptive LASSO) is asymptotic unbiased and has oracle properties [1]. We first extend *L_p _*for individual gene identification to group *L_p _*penalty for pathway selection, and then develop a novel iterative gradient algorithm for penalized global AUC summary maximization (IGGAUCS). This method incorporates the genetic pathways into global AUC summary maximization and identifies survival associated pathways instead of individual genes. The tuning parameters are determined using 10-fold cross validation with training data only. The prediction performance is evaluated using test data. We apply the proposed method to survival outcome analysis with gene expression profile and identify multiple pathways simultaneously. Experimental results with simulation and gene expression data demonstrate that the proposed procedures can be used for identifying important biological pathways that are related to survival phenotype and for building a parsimonious model for predicting the survival times.

## Background

Biologically complex diseases such as cancer are caused by mutations in biological pathways or functional groups instead of individual genes. Statistically, genes sharing the same pathway have high correlations and form functional groups or biological pathways. Many databases about biological knowledge or pathway information are available in the public domain after many years of intensive biomedical research. Such databases are often named metadata, which means data about data. Examples of such databases include the gene ontology (GO) databases (Gene Ontology Consortium, 2001), the Kyoto Encyclopedia of Genes and Genomes (KEGG) database [2], and several other pathways on the internet (e.g., http://www.superarray.com; http://www.biocarta.com). Most current methods, however, are developed purely from computational points without utilizing any prior biological knowledge or information. Gene selections with survival outcome data in the statistical literature are mainly within the penalized Cox or additive risk regression framework [3-8]. The *L*_1 _and *L_p _*(*p *< 1) penalized Cox regressions can work for simultaneous individual gene selection and survival prediction and have been extensively studied in statistics and bioinformatics literature [8-11]. The performance of the survival model is evaluated by the global area under the ROC curve summary (GAUCS) [12]. Unfortunately, those methods are mainly for individual gene selections and cannot be used to identify pathways directly. In microarray analysis, several popular tools for pathway analysis, including GENMAP, CHIPINFO, and GOMINER, are used to identify pathways that are over-expressed by differentially expressed genes. These gene set enrichment analysis (GSEA)methods are very informative and are potentially useful for identifying pathways that related to disease status [13]. One drawback with GSEA is that it considers each pathway separately and the pathway information is not utilized in the modeling stage. Wei and Li [14] proposed a boosting algorithm incorporating related pathway information for classification and regression. However, their method can only be applied for binary phenotypes. Since most complex diseases such as cancer are believed to be associated with the activities of multiple pathways, new statistical methods are required to select multiple pathways simultaneously with the time-to-event phenotypes.

A ROC curve provides complete information on the set of all possible combinations of true-positive and false-positive rates, but is also more generally useful as a graphic characterization of the magnitude of separation between the case and control distributions. AUC is known to measure the probability that the marker value (score) for a randomly selected case exceeds the marker value for a randomly selected control and is directly related to the Mann-Whitney U statistic [15,16]. In survival analysis, a survival time can be viewed as a time-varying binary outcome. Given a fixed time t, the instances for which *t_i _*= *t *are regarded as cases and samples with *t_i _*>*t *are controls. The global AUC summary (GAUCS) is then defined as *GAUCS *= *P *(*M_j _*>*M_k_*|*t_j _*<*t_k_*), which indicates that subject who died at an earlier time has a larger score value, where *M *is a score function. Heagerty and Zheng [12] have shown that GAUCS is a weighted average of the area under time-specific ROC curves. Liu et al. [17] proposed a *L*_1 _penalized quadratic support vector machine (SVM) method for GAUCS maximization and individual gene selection with survival outcomes and showed the method outperformed the Cox regression. However, that method is only for gene selections and can not be directly used for identifying pathways without additional criteria. Group LASSO (*L*_1_) related penalized methods have been extensively studied recently in logistic regression [18], multiple kernel learning [19], and microarray analysis [20] with binary phenotypes. The methods are designed for selecting groups of variables and identifying important covariate groups (pathways). However, LASSO is biased. *L_p _*(*p *≤ 1) (with one specific implementation entitled adaptive LASSO [1,21]) is asymptotic unbiased and has oracle properties. Therefore, it is reasonable to extend the *L_p _*to group *L_p _*for pathway identifications. In this paper, we therefore extend *L_p _*to group *L_p _*penalty and develop a novel iterative gradient based algorithm for GAUCS maximization (IGGAUCS), which can effectively integrate genomic data and biological pathway information and identify disease associated pathways with right censored survival data. In Section 2, we formulate the GAUCS maximization and group *L_p _*penalty model and propose an efficient EM algorithm for survival prediction. Its performance is compared with its group lasso penalized Cox regression (implemented by ourselves). We also propose an integrated algorithm with GAUCS for microarray data analysis. The proposed approach is demonstrated with simulation and gene expression examples in Section 3. Concluding remarks are discussed in Section 4.

### Group *L_p _*Penalized GAUCS Maximization

Consider we have a set of *n *independent observations ${\left\{t_{i},\delta_{i},{\text{x}}_{i}\right\}}_{i=1}^{n}$, where *δ_i _*is the censoring indicator and *t_i _*is the survival time (event time) if *δ_i _*= 1 or censoring time if *δ_i _*= 0, and **x***_i _*is the *m*-dimensional input vector of the *i*th sample. We denote $N_{i}^{*}(t)=1_{\left(t_{i}\leq t\right)}$ and the corresponding increment $dN_{i}^{*}(t)=N_{i}^{*}(t)-N_{i}^{*}(t-)$. The time-dependent sensitivity and specificity are defined by sensitivity (*c, t*): $\text{Pr}(M_{i}>c|t_{i}=t)=\text{Pr}(M_{i}>c|dN_{i}^{*}(t)\;=1)$ and specificity (*c, t*): $\left(\text{Pr}(M_{i}\leq c|t_{i}>t)=\text{Pr}(M_{i}\leq c|N_{i}^{*}(t)=0\right)$. Here sensitivity measures the expected fraction of subjects with a marker greater than *c *among the subpopulation of individuals who die (cases) at time *t*, while specificity measures the fraction of subjects with a marker less than or equal to *c *among those who survive (controls) beyond time *t*. With this definition, a subject can play the role of a control for an early time, *t *<*t_i_*, but then play the role of case when *t *= *t_i_*. Then, ROC curves are defined as *ROC_t_*(*q*) = *TP_t_*{[*FP_t_*]^-1^(*q*)} for *q *ϵ [0, 1], and the area under the ROC curve for time *t *is $AUC\left(t\right)\;\;=\;\;\int_{0}^{1}ROC_{t}(q)dq$, where *TP_t _*and *FP_t _*are the true and false positive rate at time *t *respectively, and [*FP*_*t*_]^-1^(*q*) = inf_*c*_{*c *: *FP_t_*(*c*) ≤ *q*}. ROC methods can be used to characterize the ability of a marker to distinguish cases at time *t *from controls at time *t*. However, in many applications there is no prior time *t *identified and thus a global accuracy summary is defined by averaging over *t*:

(1) $$\begin{array}{c} GAUCS\;=\;2\int AUC(t)g(t)S(t)dt\\ =\text{Pr}(M_{j}>M_{k}|t_{j}<t_{k}), \end{array}$$

which indicates the probability that the subject who died (cases) at the early time has a larger value of the marker, where *S*(*t*) and g(*t*) are the survival and corresponding density functions, respectively.

Assuming there are *r *clusters in the input covariates, our primary aim is to identify a small number of clusters associated with survival time *t_i_*. Mathematically, for each input **x***_i _*ϵ ℝ*^m^*, we are given a decomposition of ℝ*^m ^*as a product of *r *clusters:

${\mathbb{R}}^{m}={\mathbb{R}}^{m_{1}}\times ...\times {\mathbb{R}}^{m_{r}}$, so that each data point **x***_i _*can be decomposed into *r *cluster components, i.e. **x***_i _*= (**x**_*i*1_,...,**x***_ir_*), where each **x***_il _*is in general a vector. We define *M*(**x**) = **w***^T ^***x **to be the risk score function, where $\text{w}={\left({\text{w}}_{1},{\text{w}}_{2},...,{\text{w}}_{r}\right)}^{T}\in {\mathbb{R}}^{m_{1}+,...,+m_{r}}$ is the vector of coefficients that has the same cluster decomposition as **x***_i_*. We denote *M_i _*= *M*(**x***_i_*) for simplicity. Our goal is to encourage the sparsity of vector **w **at the level of clusters; in particular, we want most of its multivariate components **w***_l _*to be zero. The natural way to achieve this is to explore the combination of *L_p _*(0 ≤ *p *≤ 1) norm and *L*_2 _norm. Since **w **is defined by clusters, we define a weighted group *L_p _*norm

$$L_{p}=\sum_{l=1}^{r}d_{l}|{\text{w}}_{l}{|}^{p},$$

where within every group, an *L*_2 _norm is used $\left(|{\text{w}}_{l}|={\left({\text{w}}_{l}^{T}{\text{w}}_{l}\right)}^{1/2}\right)$ and *d_l _*can be set to be 1 if all clusters are equally important. Note that group *L_p _*= *L*_2 _if *r *= 1 and *d_l _*= 1, and group *L_p _*= *L_p _*when *r *= *m *and *d_l _*= 1. We can define the optimization problem

(2) $$\begin{array}{l} \max\;GAUCS\;=\;\max Pr(M_{j}>M_{k}|t_{j}<t_{k})\\ \text{s}.\text{t}.\;\;\;\;\;\;\;\;\;\;\;\;\;\;\;L_{p}<\beta , \end{array}$$

where *M_j _*= **w***^T ^***x***_j _*. The ideal situation is that *M*(**x***_j _*) >*M*(**x***_k _*) or **w***^T ^*(**x***_j _*- **x***_k_*) > 0, ∀ couple (**x***_j _*, **x***_k_*) with corresponding times *t_j _*<*t_k _*(or *j *<*k*) and *δ_j _*= 1.

*Pr*(*M_j _*>*M_k_|t_j _*<*t_k_*) can be estimated as

(3) $$\begin{array}{c} GAUCS\;=\;Pr(M_{j}>M_{k}|t_{j}<t_{k})\\ =\frac{\sum_{\begin{array}{l} j<k\\ \delta_{j}=1 \end{array}}\sum_{k=2}^{n}1_{M_{j}>M_{k}}}{\sum_{\begin{array}{l} j<k\\ \delta_{j}=1 \end{array}}\sum_{k=2}^{n}1}, \end{array}$$

where **1**_a>b _= 1 if *a *>*b*, and 0 otherwise. Obviously, GAUCS is a measure to rank the patients' survival time. The perfect *GAUCS *= 1 indicates that the order of all patients' survival time are predicted correctly and *GAUCS *= 0.5 indicates for a completely random choice.

One way to approximate step function $1_{M_{j}>M_{k}}$ is to use a sigmoid function $\sigma (z)=\frac{1}{1+e^{-z}}$ and let $N=\sum_{\begin{array}{l} j<k\\ \delta_{j}=1 \end{array}}\sum_{k=2}^{n}1$, then

(4) $$GAUCS=\frac{\sum_{\begin{array}{l} j<k\\ \delta_{j}=1 \end{array}}\sum_{k=2}^{n}\sigma ({\text{w}}^{T}({\text{x}}_{j}-{\text{x}}_{k}))}{N}.$$

Equation (4) is nonconvex function and can only be solved with the conjugate gradient method to find a local minimum. Based on the property that the arithmetic average is greater than the geometric average, we have

$$\frac{\sum_{\begin{array}{l} j<k\\ \delta_{j}=1 \end{array}}\sum_{k=2}^{n}\sigma ({\text{w}}^{T}({\text{x}}_{j}-{\text{x}}_{k}))}{N}\geq \frac{1}{N}\prod_{\begin{array}{l} j<k\\ \delta_{j}=1 \end{array}}\prod_{k=2}^{n}\sigma ({\text{w}}^{T}({\text{x}}_{j}-{\text{x}}_{k})).$$

We can, therefore, maximize the following log likelihood lower bound of equation (4).

(5) $$\begin{array}{c} E_{p}\;=\;\frac{1}{N}\sum_{\begin{array}{l} j<k\\ \delta j=1 \end{array}}\sum_{k=2}^{n}\log\sigma ({\text{w}}^{T}({\text{x}}_{j}-{\text{x}}_{k}))-\lambda L_{p}\\ =\frac{1}{N}\;\sum_{\begin{array}{l} j<k\\ \delta j=1 \end{array}}\sum_{k=2}^{n}\log\;\sigma ({\text{w}}^{T}({\text{x}}_{j}-{\text{x}}_{k}))-\lambda \sum_{l=1}^{r}d_{l}|{\text{w}}_{l}{|}^{p}, \end{array}$$

where λ is a penalized parameter controlling model complexity. Equation (5) is the maximum a posterior (MAP) estimator of **w **with Laplace prior provided we treat the sigmoid function as the pair-wise probability, i.e. *Pr*(*M_j _> M_k_*) = σ(**w***^T ^*(**x***_j _*- **x***_k_*)). When *p *= 1, *E_p _*is a convex function. A global optimal solution is guaranteed.

### The IGGAUCS Algorithm

In order to find the **w **that maximizes *E_p_*, we need to find the first order derivative. Since group *L_p _*with *p *≤ 1 is not differentiable at |**w***_l_*| = 0, differentiable approximations of group *L_p _*is required. We propose a local quadratic approximation for group |**w***_l_*|*^p ^*based on convex duality and local variational methods [23,24]. Fan and Li [25] proposed a similar approximation for single variable. The adaptive LASSO approach proposed by Zou and Li [21] is a LASSO (linear bound) approximation for *L_p _*penalty. The drawback with that approach is that LASSO itself is not differentiable at |**w***_l_*| = 0. Since |**w***_l_*|*^p ^*is concave when *p *< 1, we can have

(6) $$\begin{array}{c} f({\text{w}}_{l})\;=\;|{\text{w}}_{l}{|}^{p}=\underset{\eta_{l}}{\min}\{\eta_{l}|{\text{w}}_{l}{|}^{2}-g(\eta_{l})\}\\ g(\eta_{l})=\underset{\left|\theta_{l}\right|}{\min}\{\eta_{l}|\theta_{l}{|}^{2}-f(\theta_{l})\}, \end{array}$$

where the function *g*(.) is the dual function of *f*(.) in variational analysis. Geometrically, *g*(*η_l_*) represents the amounts of vertical shift applied to *η_l_*|**w***_l_*|^2 ^to obtain a quadratic upper bound with precision parameter *η_l_*, that touches *f*(**w***_l_*). Taking the first order derivative for $\eta_{l}\theta_{l}^{2}-f(\theta_{l})$, the minimum occurs at a solution of stationary equation when *θ_l _*≠ 0,

$$2\eta_{l}|\theta_{l}|-f^{\prime }(\theta_{l})=0\;\;\;\Rightarrow \;\;\;\eta_{l}=\frac{f^{\prime }(\theta_{l})}{2|\theta_{l}|},$$

and *f'*(*θ_l_*) = *p*|*θ_l_*|^*p*-1^. Substituting into *f*(**w***_l_*), we have the variational bound:

(7) $$\begin{array}{l} \left|{\text{w}}_{l}{|}^{p}\leq \frac{f^{\prime }(\theta_{l})}{2\theta_{l}}(|{\text{w}}_{l}{|}^{2}-|\theta_{l}{|}^{2})+f(\theta_{l}\right)\\ \;\;\;\;\;\;\;\;\;\;=\frac{1}{2}\{p|\theta_{l}{|}^{p-2}|{\text{w}}_{l}{|}^{2}+(2-p)|\theta_{l}{|}^{p}\}, \end{array}$$

where *θ_l _*denote variational parameters. With the local quadratic bound, we have the following smooth lower bound.

(8) $$\begin{array}{c} E_{p}\;=\;\frac{1}{N}\sum_{\begin{array}{l} j<k\\ \delta j=1 \end{array}}\sum_{k=2}^{n}\log\sigma ({\text{w}}^{T}({\text{x}}_{j}-{\text{x}}_{k}))-\lambda \sum_{l=1}^{r}d_{l}|{\text{w}}_{l}{|}^{p}\\ \geq \frac{1}{N}\sum_{\begin{array}{l} j<k\\ \delta j=1 \end{array}}\sum_{k=2}^{n}\log\sigma ({\text{w}}^{T}({\text{x}}_{j}-{\text{x}}_{k}))\\ -\lambda \sum_{l=1}^{r}\frac{d_{l}}{2}\{p|\theta_{l}{|}^{p-2}|{\text{w}}_{l}{|}^{2}+(2-p)|\theta_{l}{|}^{p}\}\\ =E(\text{w},\theta ). \end{array}$$

In equation (8), the lower bound *E*(**w**, *θ*) is differentiable w.r.t both **w **and *θ*. We therefore propose a EM algorithm to maximize *E*(**w**, *θ*) w.r.t **w **while keeping *θ *fixed and maximize *E*(**w**, *θ*) w.r.t the variational parameter *θ *to tighten the variational bound while keeping **w **fixed. Convergence to the local optimum is guaranteed. Since maximization w.r.t the variational parameters *0 *= (|*θ*_1_|,|*θ*_2_|,..., |*θ_r_*|), with **w **being fixed, can be solved with the stationary equation $\frac{\partial E(\text{w},\theta )}{\partial |\theta_{l}|}=0$ we have *θ_l _*= **w***_l_*, for *l *= 1, 2,..., *r*.

Given *r *candidate pathways potentially associated with the survival time, *m_l _*survival associated genes with the expression of **x***_l _*on each pathway *l *(*l *= 1, 2,..., *r*), and letting **w **= (**w**_1_, **w**_2_,..., **w***_r_*)^*T *^be a vector of the corresponding coefficients and $g(\text{w})=\frac{\partial_{\text{w}}E(\text{w},\theta )}{\partial \text{w}}$, we have the following iterative gradient algorithm for *E*(**w**, *θ*) maximization:

### The IGGAUCS Algorithm

Given *p*, λ, and *ϵ *= 10^-6^, initializing ${\text{w}}^{1}={\left({\text{w}}_{1}^{1},{\text{w}}_{2}^{1},...,{\text{w}}_{r}^{1}\right)}^{T}$ randomly with nonzero ${\text{w}}_{l}^{1},\;l=1,...,\;r$, and set *θ*^1 ^= w^1^.

Update w with *θ *fixed:

**w**^*t*+1 ^= **w**^*t *^+ *α^t^d^t^*, where *t*: the number of iterations and *α^t^*: the step size, *d^t ^*is updated with the conjugate gradient method:

*d^t ^*= g(**w**^*t*^) + *u^t^d^t ^*and $u^{t}=\frac{{\left[g({\text{w}}^{t})-g({\text{w}}^{t-1})\right]}^{T}g({\text{w}}^{t})}{g{\left({\text{w}}^{t-1}\right)}^{T}g({\text{w}}^{t-1})}$.

Update θ with w fixed:

*θ*^*t*+1 ^= **w**^*t*+1^

Stop when |**w**^*t*+1 ^- **w**^*t*^| <*ϵ *or maximal number of iterations exceeded.

### Choice of Parameters

There are two parameters *p *and λ in this method, which can be determined through 10-fold cross validation. One efficient way is to set *p *= 0.1, 0.2,..., and 1 respectively, and search for an optimal λ for each *p *using cross validation. The best (*p*, λ) pair will be found with the maximal test GAUCS value. Theoretically when *p *= 1, *E*(**w**, *θ*) is convex and we can find the global maximum easily, but the solution is biased and small values of p would lead to better asymptotic unbiased solutions. Our results with limited experiments show that optimal *p *usually happens at a small *p *such as *p *= 0.1. For comparison purposes, we implement the popular Cox regression with group LASSO (G*L*_1_Cox), since there is no software available in the literature. Our implementation is based on group LASSO penalized partial log-likelihood maximization. The best λ is searched from λ ϵ [0.1, 25] for IGGAUCS and from λ ϵ [0.1, 40] for G*L*_1_Cox method with the step size of 0.1, as the *L_p _*penalty goes to zero much quicker than *L*_1_. We suggest that the larger step size such as 0.5 can be used for most applications, since the test GAUCS does not change dramatically with a small change of λ.

## Computational Results

### Simulation Data

We first perform simulation studies to evaluate how well the IGGAUCS procedure performs when input data has a block structure. We focus on whether the important variable groups that are associated with survival outcomes can be selected using the IGGAUCS procedure and how well the model can be used for predicting the survival time for future patients. In our simulation studies, we simulate a data set with a sample size of 100 and 300 input variables with 100 groups (clusters). The triple variables **x**_1 _- **x**_3_, **x**_4 _- **x**_6_, **x**_7 _- **x**_9_,..., **x**_298 _- **x**_300 _within each group are highly correlated with a common correlation γ and there are no correlations between groups. We set γ = 0.1 for weak correlation, γ = 0.5 for moderate, and γ = 0.9 for strong correlation in each triple group and generate training and test data sets of sample size 100 with each γ respectively from a normal distribution with the band correlation structure. We assume that the first three groups(9 covariates) (**x**_1 _- **x**_3_, **x**_4 _- **x**_6_, **x**_7 _- **x**_9_) are associated with survival and the 9 covariates are set to be **w **= [-2.9 2.1 2.4 1.6 -1.8 1.4 0.4 0.8 -0.5]^*t*^. With this setting, 3 covariates in the first group have the strongest association (largest covariate values) with survival time and 3 covariates in group 3 have less association with survival time. The survival time is generated with *H *= 100 exp(-**w**^*T *^**x **+ *ε*) and the Weibull distribution, and the census time is generated from 0.8*median(time) plus a random noise. Based on this setting, we would expect about 25% - 35% censoring. To compare the performance of IGGAUCS and G*L*_1_Cox, we build the model based on training data set and evaluate the model with the test data set. We repeat this procedure 100 times and use the time-independent GAUCS to assess the predictive performance.

We first compare the performance of IGGAUCS and G*L*_1_Cox methods with the frequency of each of these three groups being selected under two different correlation structures based on 100 replications. The results are in Table 1. Table 1 shows that IGGAUCS with *p *= 0.1 outperforms the G*L*_1_Cox in that IGGAUCS can identify the true group structures more frequently under different inner group correlation structures. Its performance is much better than G*L*_1_Cox regression, when the inner correlation in a group is high (γ = 0.9) and the variables within a group have weak association with survival time.

**Table 1:** Frequency of Three Survival Associated Groups Selected in 100 Replications

	**IGGAUCS/*GL***_**1**_**Cox**		
Parameters	γ = 0.1	γ = 0.5	γ = 0.9
*w*_1 _= -2.9			
*w*_2 _= 2.1	100/100	100/100	100/100
*w*_3 _= 2.4			

*w*_4 _= 1.6			
*w*_5 _= -1.8	100/78	100/84	100/96
*w*_6 _= 1.4			

*w*_7 _= 0.4			
*w*_8 _= 0.8	47/2	53/4	94/24
*w*_9 _= -0.5			

**Figure 1:**
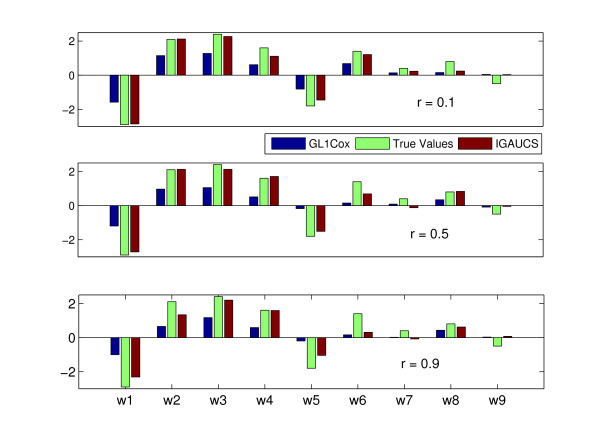
**True and Estimated Parameters**. The true and estimated parameters with the simulation data are shown in Figure 2. The left bars represent each parameter estimated from *GL*_1_Cox, the middle bars are the true value of the parameter, and the right bars indicate parameters estimated form IGGAUCS.

To compare more about the performance of IGGAUCS and G*L*_1_Cox in parameter estimation, we show the results for each parameter with different inner correlation structures (0.1, 0.5, 0.9) in Figure 1. For each parameter in Figure 1, the left bar represents the parameter estimated from G*L*_1_Cox, the middle bar is the true value of the parameter, and the right bar indicates parameter estimated from IGGAUCS. We observe that both the G*L*_1_Cox and IGGAUCS methods estimated the sign of the parameters correctly for the first two groups. However both methods can only estimate the sign of *w*_8 _correctly in group 3 with smaller coefficients. Moreover, **ŵ **estimated from IGGAUCS is much closer to the true **w **than that from G*L*_1_Cox, especially when the covariates are larger. This indicates that the *L_p _*(*p *= 0.1) penalty is less biased than the *L*_1 _penalty. The estimators of IGGAUCS are larger than that of G*L*_1_Cox with weak, moderate, and strong correlations. Finally, the test global AUC summaries (GAUCSs) of IGGAUCS and G*L*1Cox with 100 replications are shown in Table 2. Table 2 shows that IGGAUCS performs better than G*L*_1_Cox regression. This is reasonable, since our method, unlike Cox regression which maximizes a partial log likelihood, directly maximizes the penalized GAUCS. One interesting result is that the test GAUCSs become smaller as the inner group correlation coefficient γ increases from 0.1 to 0.9. We also apply the gene harvesting method proposed by Hastie et al. (2001) and discussed by Segal (2006) [7,26] to the simulation data, but don't show the results in Table 2. The gene harvest method uses the average gene expression in each group (cluster) and ignore the variance among genes within the group. The prediction performances are poor with test GAUCS of 0.57 ± 0.02 and 0.65 ± 0.016 respectively, when the correlations among genes are weak (γ = 0.1) and moderate (γ = 0.5). Its performance is slightly better with the test GAUCS of 0.75 ± 0.024, when γ = 0.9, but this4 performance is still not as good as either IGGAUCS or *GL*_1_Cox. One explantation is that the group is more heterogeneous with weaker correlations among variables, and the average does not provide a meaningful summary. Moreover, we cannot identify the survival association of individual variables using gene harvesting.

**Table 2:** Test GAUCS of Simulated Data *w*ith Different Correlation Structures

Correlation	IGGAUCS	***GL***_**1**_**Cox**
γ = 0.1	0.921(±0.023)	0.897(±0.031)
γ = 0.5	0.889(±0.021)	0.871(±0.024)
γ = 0.9	0.866(±0.017)	0.828(±0.025)

#### Follicular Lymphoma (FL) Data

Follicular lymphoma is a common type of Non-Hodgkin Lymphoma (NHL). It is a slow growing lymphoma that arises from B-cells, a type of white blood cell. It is also called an "indolent" or "low-grad" lymphoma for its slow nature, both in terms of its behavior and how it looks under the microscope. A study was conducted to predict the survival probability of patients with gene expression profiles of tumors at diagnosis [27].

Fresh-frozen tumor biopsy specimens and clinical data were obtained from 191 untreated patients who had received a diagnosis of follicular lymphoma between 1974 and 2001. The median age of patients at diagnosis was 51 years (range 23 - 81) and the median follow up time was 6.6 years (range less than 1.0 - 28.2). The median follow up time among patients alive was 8.1 years. Four records with missing survival information were excluded from the analysis. Affymetrix U133A and U133B microarray gene chips were used to measure gene expression levels from RNA samples. A log 2 transformation was applied to the Affymetrix measurement. Detailed experimental protocol can be found in Dave et al. 2004. The data set was normalized for each gene to have mean 0 and variance 1. Because the data is very large and there are many genes with their expressions that either do not change cross samples or change randomly, we filter out the genes by defining a correlation measure with GAUCS for each gene **x**_i _*R*(*t*, **x***_i_*) = |2*GAUCS*(*t*, **x***_i_*) - 1|, where *R *= 1 when *GAUCS *= 0, or 1 and *R *= 0 when *GAUCS *= 0.5 (gene **x***_i _*is not associated with the survival time). We perform the permutation test 1000 times for *R *to identify 2150 probes associated with survival time. We then identify 49 candidate pathways with 5 and more genes using DAVID. There are total 523 genes on the candidate pathways. Since a gene can be involved in more than one pathway, the number of distinguished genes should be a little less than 500. The 49 biological pathways are given in Table 3. We finally apply IGGAUCS to identify the small number of biological pathways associated with survival phenotypes. We first randomly divide the data into two subsets, one for training with 137 samples, and the other for testing with 50 samples. To avoid overfitting and bias from a particular partition, we randomly partition the data 50 times to estimate the performance of the model with the average of the test GAUCS. The regularization parameter λ is tuned using 10-fold cross-validation with training data only.

**Table 3:** Candidate Survival Associated Pathways

Pathways	# of Genes	Pathways	# of Genes
Propanoate metabolism	5	Melanoma	10
Type II diabetes mellitus	6	Thyroid cancer	5
Adipocytokine signaling pathway	10	Prostate cancer	13
Melanogenesis	13	Glycolysis/Gluconeogenesis	8
GnRH signaling pathway	11	Butanoate metabolism	6
Insulin signaling pathway	15	Endometrial cancer	11
Sphingolipid metabolism	5	Pancreatic cancer	10
Glycerophospholipid metabolism	9	Colorectal cancer	12
T cell receptor signaling pathway	11	RNA polymerase	6
Hematopoietic cell lineage	10	Huntington's disease	5
Glycerolipid metabolism	6	Focal adhesion	20
Toll-like receptor signaling pathway	13	Apoptosis	12
Antigen processing and presentation	9	Adherens junction	10
Complement and coagulation cascades	8	Tryptophan metabolism	7
ECM-receptor interaction	14	Histidine metabolism	6
Wnt signaling pathway	20	Fatty acid metabolism	10
Ubiquitin mediated proteolysis	15	Acute myeloid leukemia	9
Neuroactive ligand-receptor interaction	28	Bladder cancer	6
gamma-Hexachlorocyclohexane degradation	5	Focal adhesion	20
Calcium signaling pathway	21	ErbB signaling pathway	11
MAPK signaling pathway	31	PPAR signaling pathway	16
Valine, leucine and isoleucine degradation	6	Glioma	7
Pyrimidine metabolism	12	Chronic myeloid leukemia	10
Glycan structures - degradation	5	Non-small cell lung cancer	11
Porphyrin and chlorophyll metabolism	7		

Since it is possible different pathways may be selected in the cross validation procedure, the relevance count concept [28] was utilized to count how many times a pathway is selected in the cross validation. Clearly, the maximum relevance count for a pathway is 200 with the 10-fold cross validation and 20 repeating. We have selected 8 survival associated pathways with IGGAUCS. The average test GAUCS is 0.892 ± 0.013. Moreover, the parameters (weights) **w***_i _*and corresponding genes on each pathway indicate the association strength and direction between genes and the survival time. Positive *w_i_*s indicate that patients with high expression level die earlier and negative *w_i_*s represent that patients live longer with relatively high expression levels. The absolute values of |*w_i_*| indicate the strength of association between survival time and the specific gene. Genes on the pathway, estimated parameters, and relevance accounts are given in Table 4.

**Table 4:** Genes on pathway, relevance accounts, and estimated parameters

*w_i_*	GeneID	Gene Name
**ECM-receptor interaction (relevance counts: 200)**		

0.2239	CD36	cd36 antigen (collagen type i receptor, thrombospondin receptor)
0.0409	FNDC1	fibronectin type iii domain containing 1
0.0746	SV2C	synaptic vesicle glycoprotein 2c
0.0804	SDC1	syndecan 1
-0.1255	FN1	fibronectin 1
0.0211	LAMC1	laminin, gamma 1 (formerly lamb2)
-0.0854	GP5	glycoprotein v (platelet)
-0.1130	CD47	cd47 antigen (rh-related antigen, integrin-associated signal transducer)
-0.1296	THBS2	thrombospondin 2
-0.0547	COL1A2	collagen, type i, alpha 2
-0.1024	COL5A2	collagen, type v, alpha 2
0.0861	LAMB4	laminin, beta 4
-0.0315	COL1A1	collagen, type i, alpha 1
0.0395	AGRN	agrin RG

**Focal adhesion (relevance counts 145)**		

0.0054	PAK3	p21 (cdkn1a)-activated kinase 3
0.0446	PIK3R3	phosphoinositide-3-kinase, regulatory subunit 3 (p55, gamma)
0.0044	PDPK1	3-phosphoinositide dependent protein kinase-1
-0.0045	BAD	bcl2-antagonist of cell death
0.0087	PARVA	parvin, alpha
-0.0144	FN1	fibronectin 1
0.0041	LAMC1	laminin, gamma 1 (formerly lamb2)
-0.0202	PARVG	parvin, gamma
-0.0158	THBS2	thrombospondin 2
0.0134	PPP1R12A	protein phosphatase 1, regulatory (inhibitor) subunit 12a
-0.0044	SOS1	son of sevenless homolog 1 (drosophila)
-0.0084	COL1A2	collagen, type i, alpha 2
-0.0122	COL5A2	collagen, type v, alpha 2
0.0091	LAMB4	laminin, beta 4 RG Homo sapiens
-0.0068	RAF1	v-raf-1 murine leukemia viral oncogene homolog 1
-0.0038	ACTN1	actinin, alpha 1
-0.0034	COL1A1	collagen, type i, alpha 1
-0.0067	GSK3B	glycogen synthase kinase 3 beta
-0.0065	MAPK8	mitogen-activated protein kinase 8
-0.0030	MYL7	myosin, light polypeptide 7, regulatory

**Neuroactive ligand-receptor interaction (relevance counts: 200)**		

0.0894	P2RY6	pyrimidinergic receptor p2y, g-protein coupled, 6
-0.2753	PTAFR	platelet-activating factor receptor
0.1648	GLRA3	glycine receptor, alpha 3
0.0857	FPRL1	formyl peptide receptor-like 1
-0.1783	EDNRA	endothelin receptor type a
0.3233	HRH4	histamine receptor h4
0.2106	GRM2	glutamate receptor, metabotropic 2
-0.1112	GRIN1	glutamate receptor, ionotropic, n-methyl d-aspartate 1
-0.0220	PTHR1	parathyroid hormone receptor 1
0.0971	OPRM1	opioid receptor, mu 1
-0.4303	CTSG	cathepsin g
-0.0404	P2RY8	purinergic receptor p2y, g-protein coupled, 8
-0.0783	BDKRB1	bradykinin receptor b1
0.3247	FSHR	follicle stimulating hormone receptor
-0.1430	ADRA1B	adrenergic, alpha-1b-, receptor
0.1464	C3AR1	complement component 3a receptor 1
0.1120	P2RX2	purinergic receptor p2x, ligand-gated ion channel, 2
0.0311	AVPR1B	arginine vasopressin receptor 1b
0.2646	FPR1	formyl peptide receptor 1
0.2003	GABRA5	gamma-aminobutyric acid (gaba) a receptor, alpha 5
-0.0278	PRLR	prolactin receptor
-0.1070	ADORA1	adenosine a1 receptor
0.2652	HTR7	5-hydroxytryptamine (serotonin) receptor 7 (adenylate cyclase-coupled)
-0.0194	GABRA4	gamma-aminobutyric acid (gaba) a receptor, alpha 4
0.0145	GHRHR	growth hormone releasing hormone receptor
-0.3163	MAS1	mas1 oncogene
-0.0760	PTGER3	prostaglandin e receptor 3 (subtype ep3)
0.2196	PARD3	par-3 partitioning defective 3 homolog (c. elegans)

**Ubiquitin mediated proteolysis (relevance counts: 200)**		

0.0186	UBE2B	ubiquitin-conjugating enzyme e2b (rad6 homolog)
-0.0677	CUL4A	cullin 4a
0.0051	PML	promyelocytic leukemia
-0.1023	UBE3B	ubiquitin protein ligase e3b
-0.1581	UBE3C	ubiquitin protein ligase e3c
0.1390	BTRC	beta-transducin repeat containing
-0.0669	HERC3	hect domain and rld 3
0.00009	RBX1	ring-box 1
0.0011	CUL5	cullin 5
0.0267	ANAPC4	anaphase promoting complex subunit 4
-0.0253	UBE2L3	ubiquitin-conjugating enzyme e2l 3
0.0096	KEAP1	kelch-like ech-associated protein 1
-0.0267	UBE2E1	ubiquitin-conjugating enzyme e2e 1 (ubc4/5 homolog, yeast)
0.0116	CBL	cas-br-m (murine) ecotropic retroviral transforming sequence
0.0328	BIRC6	baculoviral iap repeat-containing 6 (apollon)

**Porphyrin and chlorophyll metabolism (relevance counts: 185)**		

0.0330	BLVRA	biliverdin reductase a
-0.0103	FTH1	ferritin, heavy polypeptide 1
0.0227	ALAD	aminolevulinate, delta-, dehydratase
0.1983	HMOX1	heme oxygenase (decycling) 1
0.0070	UROS	uroporphyrinogen iii synthase (congenital erythropoietic porphyria)
-0.1596	GUSB	glucuronidase, beta
0.0077	UGT2B15	udp glucuronosyltransferase 2 family, polypeptide b15

**Calcium signaling pathway (relevance counts: 200)**		

-0.0025	BST1	bone marrow stromal cell antigen 1
0.0003	BDKRB1	bradykinin receptor b1
-0.0016	PTAFR	platelet-activating factor receptor
-0.0002	ADRA1B	adrenergic, alpha-1b-, receptor
-0.0007	PPP3CC	protein phosphatase 3 (formerly 2b), catalytic subunit, gamma isoform
-0.0001	ADCY7	adenylate cyclase 7
0.0008	GNA11	guanine nucleotide binding protein (g protein), alpha 11 (gq class)
-0.0013	AVPR1B	arginine vasopressin receptor 1b
0.0014	P2RX2	purinergic receptor p2x, ligand-gated ion channel, 2
-0.0013	CACNA1E	calcium channel, voltage-dependent, alpha 1e subunit
0.0004	EDNRA	endothelin receptor type a
0.00009	SLC8A1	solute carrier family 8 (sodium/calcium exchanger), member 1
0.0006	CACNA1B	calcium channel, voltage-dependent, l type, alpha 1b subunit
-0.0013	PLCD1	phospholipase c, delta 1
-0.0029	HTR7	5-hydroxytryptamine (serotonin) receptor 7 (adenylate cyclase-coupled)
0.0014	GRIN1	glutamate receptor, ionotropic, n-methyl d-aspartate 1
0.0028	CAMK2A	calcium/calmodulin-dependent protein kinase (cam kinase) ii alpha
-0.0024	CACNA1I	calcium channel, voltage-dependent, alpha 1i subunit
-0.0002	TNNC1	troponin c type 1 (slow)
0.0009	PTGER3	prostaglandin e receptor 3 (subtype ep3)
0.0012	CACNA1F	calcium channel, voltage-dependent, alpha 1f subunit

**Fatty acid metabolism (relevance counts: 192)**		

0.0043	ACSL3	acyl-coa synthetase long-chain family member 3
0.0675	ALDH2	aldehyde dehydrogenase 2 family (mitochondrial)
-0.0491	ACAT2	acetyl-coenzyme a acetyltransferase 2 (acetoacetyl coenzyme a thiolase)
0.0120	ALDH1B1	aldehyde dehydrogenase 1 family, member b1
0.0597	CYP4A11	cytochrome p450, family 4, subfamily a, polypeptide 11
-0.1447	ACADSB	acyl-coenzyme a dehydrogenase, short/branched chain
0.0736	CPT1A	carnitine palmitoyltransferase 1a (liver)
0.1075	CPT1B	carnitine palmitoyltransferase 1b (muscle)
-0.0366	ACADVL	acyl-coenzyme a dehydrogenase, very long chain
0.2168	ADH4	alcohol dehydrogenase 4 (class ii), pi polypeptide

**MAPK signaling pathway (relevance counts: 200)**		

-0.1570	PLA2G10	phospholipase a2, group x
-0.2415	MAPKAPK5	mitogen-activated protein kinase-activated protein kinase 5
-0.1636	IL1B	interleukin 1, beta
-0.0651	ZAK	sterile alpha motif and leucine zipper containing kinase azk
0.0572	PPP3CC	protein phosphatase 3 (formerly 2b), catalytic subunit, gamma isoform
-0.0674	MAP3K2	mitogen-activated protein kinase kinase kinase 2
-0.1729	JUND	jun d proto-oncogene
-0.1718	SOS1	son of sevenless homolog 1 (drosophila)
0.1082	FGF14	fibroblast growth factor 14
0.3102	PTPN5	protein tyrosine phosphatase, non-receptor type 5
-0.3903	CACNB1	calcium channel, voltage-dependent, beta 1 subunit
0.2678	MAP3K7	mitogen-activated protein kinase kinase kinase 7
0.3176	CACNG8	calcium channel, voltage-dependent, gamma subunit 8
0.0893	FGF19	fibroblast growth factor 19
-0.0853	RRAS2	related ras viral (r-ras) oncogene homolog 2
0.0215	NLK	nemo-like kinase
0.0452	MAP4K4	mitogen-activated protein kinase kinase kinase kinase 4
0.1639	CACNA1E	calcium channel, voltage-dependent, alpha 1e subunit
-0.0290	ARRB1	arrestin, beta 1
-0.1169	STK4	serine/threonine kinase 4
0.1008	CACNA1B	calcium channel, voltage-dependent, l type, alpha 1b subunit
0.0839	MOS	v-mos moloney murine sarcoma viral oncogene homolog
-0.1244	MEF2C	mads box transcription enhancer factor 2, polypeptide c
0.1572	RAF1	v-raf-1 murine leukemia viral oncogene homolog 1
0.1757	MAPK8IP1	mitogen-activated protein kinase 8 interacting protein 1
0.2908	IKBKB	inhibitor of kappa light polypeptide gene enhancer in b-cells, kinase beta
-0.3452	CACNA1I	calcium channel, voltage-dependent, alpha 1i subunit
-0.2473	MAPK8	mitogen-activated protein kinase 8
-0.2339	CACNA1F	calcium channel, voltage-dependent, alpha 1f subunit
0.0979	CD14	cd14 antigen
-0.1047	MRAS	muscle ras oncogene homolog

**Figure 2:**
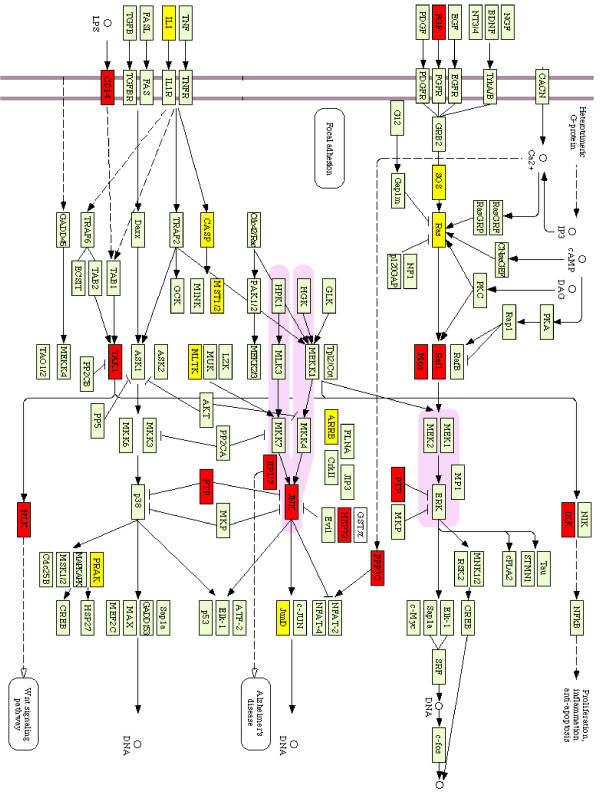
**MAPK Signaling Pathway**. MAPK signaling pathway and the associated genes. Genes in red are highly expressed in patients who died earlier and genes in yellow are highly expressed in patients who lived longer.

The eight KEGG pathways identified play an important role in patient survivals and they can be ranked with the average |*w_j _*|:*∑_j _*|*w_j _*|/*L*, where *L *is the number of genes on a pathway as shown in Table 5. The identified 8 KEGG pathways fall into three categories (i) signaling molecules and interaction including the MAPK signaling pathway, the Calcium signaling pathway, Focal adhesion, ECM-receptor interactions and neuroactive ligand-receptor interactions, (ii) metabolic pathways including Fatty acid metabolism and Porphyrin and chlorophyll metabolism, and (iii) nitric oxide and cell stress including Ubiquitin mediated proteolysis. These pathways are involved in different aspects of genetic functions and are vital for cancer patient survivals. We only discuss the MAPK signaling pathway but others can be analyzed in a similar fashion. The top-rank MAPK signaling pathway transduces a large variety of external signals, leading to a wide range of cellular responses, including growth, differentiation, inflammation, and apoptosis invasiveness and ability to induce neovascularization. MAPK signaling pathway has been linked to different cancers including follicular lymphoma [29]. The pathway and genes on pathways are given in Figure 2.

**Table 5:** Pathway Ranks

Pathway	***∑***_***j ***_**|*w***_***j***_**|/*L***	Rank
MAPK signaling pathway	0.1614	1
Neuroactive ligand-receptor interaction	0.1562	2
ECM-receptor interaction	0.0863	3
Fatty acid metabolism	0.0772	4
Porphyrin and chlorophyll metabolism	0.0627	5
Ubiquitin mediated proteolysis	0.0494	6
Focal adhesion	0.0100	7
Calcium signaling pathway	0.0012	8

Genes in red color are highly expressed in patients with aggressive FL and genes in yellow are highly expressed in the earlier stage of FL cancers. Many important cancer related genes are identified with our methods. For example, SOS1, one of the RAS genes (e.g., MIM 190020), encodes membrane-bound guanine nucleotide-binding proteins that function in the transduction of signals that control cell growth and differentiation. Binding of GTP activates RAS proteins, and subsequent hydrolysis of the bound GTP to GDP and phosphate inactivates signaling by these proteins. GTP binding can be catalyzed by guanine nucleotide exchange factors for RAS, and GTP hydrolysis can be accelerated by GTPase-activating proteins (GAPs). SOS1 plays a crucial role in the coupling of RTKs and also intracellular tyrosine kinases to RAS activation. The deregulation of receptor tyrosine kinases (RTKs) or intracellular tyrosine kinases coupled to RAS activation has been involved in the development of a number of tumors, such as those in breast cancer, ovarian cancer and leukemia. Another gene, IL1B, is one of a group of related proteins made by leukocytes (white blood cells) and other cells in the body. IL1B, one form of IL1, is made mainly by one type of white blood cell, the macrophage, and helps another type of white blood cell, the lymphocyte, fight infections. It also helps leukocytes pass through blood vessel walls to sites of infection and causes fever by affecting areas of the brain that control body temperature. IL1B made in the laboratory is used as a biological response modifier to boost the immune system in cancer therapy.

As shown in Figure 2, the genes SOS1, IL1B, RAS, CACNB1, MEF2C, JUND, and MAPKAPK5 are highly expressed in patients who were diagnosed earlier and lived longer and the genes FGF14, PTPN5, MOS, RAF1, CD14 are highly expressed in patients who were diagnosed at more aggressive stages and died earlier, which may indicate that oncogenes such such SOS1, JUND, and RAS may initialize FL cancer and genes such as MOS, IKK, and CD14 may cause FL cancer to be more aggressive. There are several causal relations among the identified genes on MAPK. For instance, the down-expressed SOS and RAS cause the up-expressed RAF1 and MOS and the up-stream gene IL1 is coordinately expressed with CASP and the gene MST1/2.

## Conclusions

Since a large amount of biological information on various aspects of systems and pathways is available in public databases, we are able to utilize this information in modeling genomic data and identifying pathways and genes and their interactions that might be related to patient survival. In this study, we have developed a novel iterative gradient algorithm for group *L_p _*penalized global AUC summary (IGGAUCS) maximization methods for gene and pathway identification, and for survival prediction with right censored survival data and high dimensional gene expression profile. We have demonstrated the applications of the proposed method with both simulation and the FL cancer data set. Empirical studies have shown the proposed approach is able to identify a small number of pathways with nice prediction performance. Unlike traditional statistical models, the proposed method naturally incorporates biological pathways information and it is also different from the commonly used Gene Set Enrichment Analysis (GSEA) in that it simultaneously considers multiple pathways associated with survival phenotypes.

With comprehensive knowledge of pathways and mammalian biology, we can greatly reduce the hypothesis space. By knowing the pathway and the genes that belong to particular pathways, we can limit the number of genes and gene-gene interactions that need to be considered in modeling high dimensional microarray data. The proposed method can efficiently handle thousands of genes and hundreds of pathways as shown in our analysis of the FL cancer data set.

There are several directions for our future investigations. For instance, we may want to further investigate the sensitivity of the proposed methods to the misspecification of the pathway information and misspecification of the model. We may also extend our method to incorporate gene-gene interactions and gene (pathway)- environmental interactions.

Even though we have only applied our methods to gene expression data, it is straightforward to extend our methods to SNP, miRNA CGH, and other genomic data without much modification.

## Competing interests

The authors declare that they have no competing interests.

## Authors' contributions

ZL designed the method and drafted the manuscript. Both LSM and TH participated manuscript preparation and revised the manuscript critically. LM provided important help in its biological contents. All authors read and approved the final manuscript.
